# Comparative Metabolome and Transcriptome Analysis of Rapeseed (*Brassica napus* L.) Cotyledons in Response to Cold Stress

**DOI:** 10.3390/plants13162212

**Published:** 2024-08-09

**Authors:** Xinhong Liu, Tonghua Wang, Ying Ruan, Xiang Xie, Chengfang Tan, Yiming Guo, Bao Li, Liang Qu, Lichao Deng, Mei Li, Chunlin Liu

**Affiliations:** 1 Crop Research Institute, Hunan Academy of Agricultural Sciences, Changsha 410125, China; xinhong1220@hunaas.cn (X.L.); guoyiming@hunaas.cn (Y.G.);; 2 Key Laboratory of Hunan Provincial on Crop Epigenetic Regulation and Development, Hunan Agricultural University, Changsha 410128, China; yingruan@hotmail.com (Y.R.); xie18773658581@163.com (X.X.); tanchengfang@hunau.edu.cn (C.T.); 3 Yuelushan Laboratory, Hunan Academy of Agricultural Sciences, Changsha 410125, China; 4 College of Agronomy, Hunan Agricultural University, Changsha 410128, China

**Keywords:** cold sensitive, cell wall modification, ion homeostasis, phytohormone responsive genes, reactive oxygen species, sugar biosynthesis, electron transfer chain

## Abstract

Cold stress affects the seed germination and early growth of winter rapeseed, leading to yield losses. We employed transmission electron microscopy, physiological analyses, metabolome profiling, and transcriptome sequencing to understand the effect of cold stress (0 °C, LW) on the cotyledons of cold-tolerant (GX74) and -sensitive (XY15) rapeseeds. The mesophyll cells in cold-treated XY15 were severely damaged compared to slightly damaged cells in GX74. The fructose, glucose, malondialdehyde, and proline contents increased after cold stress in both genotypes; however, GX74 had significantly higher content than XY15. The pyruvic acid content increased after cold stress in GX74, but decreased in XY15. Metabolome analysis detected 590 compounds, of which 32 and 74 were differentially accumulated in GX74 (CK vs. cold stress) and XY15 (CK vs. cold stressed). Arachidonic acid and magnoflorine were the most up-accumulated metabolites in GX74 subjected to cold stress compared to CK. There were 461 and 1481 differentially expressed genes (DEGs) specific to XY15 and GX74 rapeseeds, respectively. Generally, the commonly expressed genes had higher expressions in GX74 compared to XY15 in CK and cold stress conditions. The expression changes in DEGs related to photosynthesis-antenna proteins, chlorophyll biosynthesis, and sugar biosynthesis-related pathways were consistent with the fructose and glucose levels in cotyledons. Compared to XY15, GX74 showed upregulation of a higher number of genes/transcripts related to arachidonic acid, pyruvic acid, arginine and proline biosynthesis, cell wall changes, reactive oxygen species scavenging, cold-responsive pathways, and phytohormone-related pathways. Taken together, our results provide a detailed overview of the cold stress responses in rapeseed cotyledons.

## 1. Introduction

Rapeseed (*Brassica napus* L.) is the fourth largest crop in China after rice, maize, and wheat. It is mainly cultivated for human as well as animal consumption i.e., for edible oil and oil seed cake. Moreover, it is also used for non-edible purposes in the industries involved in biodiesel, lubricants, and plastic [1]. China is the world’s second largest rapeseed producer, with an area of 7253.47 thousand hectares and a production of 15.5314 million tons in 2022 (https://www.chyxx.com/industry/1188674.html; accessed on 5 August 2024). There are two types of rapeseeds, i.e., winter and spring rapeseed, with the former accounting for more than 90% of the crop grown in China. Its sowing in October–November exposes seedlings to 0–4 °C, due to which the overall crop suffers. Nearly 77.8% of the rapeseed producing areas in China are cold-affected. Yield reductions of nearly 11% have been reported due to cold stress [2]. Additionally, the cultivation of rice crops with different ripening times in the Yangtze River Basin postpones the sowing date of rapeseed to late October–early November. This exposes rapeseed to low temperatures. The concomitant increase in import volumes of rapeseed oil and meal during the last five years (https://www.statista.com/; accessed on 8 November 2022) indicates that China will need to improve its rapeseed area and production. To expand the rapeseed industry, it has been suggested that it can be cultivated in rotation with crops in cold and arid regions [3]. Therefore, to survive in cold conditions, cold tolerance mechanisms should be identified so that cold-tolerant genotypes can be developed.

Seed germination, early growth, and the subsequent establishment of the seedlings are pivotal for a successful crop. A key stage during seed germination and post-germinative growth is the transition from the heterotrophic to the autotrophic stage. This phase transition is highly vulnerable to abiotic stresses [4]. As indicated above, winter rapeseed is exposed to 0–4 °C during the germination period in the field; therefore, the phase transition and the survival of the young cotyledons are highly associated with a successful stand establishment and overall yield [5]. Studies have shown that cold stress can negatively affect cotyledons by inducing the production of reactive oxygen species (ROS). Moreover, cold stress also damages the cell membrane, reducing the efficiency of photosynthesis, which is why the plants may attain an abnormal morphology or exhibit retarded growth [6,7]. Furthermore, cold stress can also affect the enzyme activities involved in sucrose and starch partitioning [8]. Generally, cold stress has been shown to cause dehydration, marked wilting, and chlorosis [9,10]. Calluses of rapeseed genotypes with contrasting cold sensitivity exhibited physiological changes, including in relative electrolyte leakage, ROS generation, soluble sugars and proteins, malondialdehyde (MDA), inositol, and proline content [11]. Cold stress also changes the activities of the ROS-scavenging enzymes [9]. Our earlier investigation of rapeseed genotypes with contrasting cold sensitivity has shown that cold stress can significantly impair silique length in cold-sensitive genotypes compared to tolerant ones. Similarly, relative soluble sugar content and peroxidase activity (POD) [12] in sensitive genotypes showed limited increases after cold stress, whereas the cold-tolerant genotype exhibited a sharp and significant increase in soluble sugar content and POD activity. Nevertheless, almost all the information that is available on the cold stress responses in rapeseed are limited to calluses, fully developed seedlings e.g., 21 days old, mature plant stages, or mature plant tissues e.g., leaves or siliques [9,10,11,13]. The knowledge about the key physiological responses of rapeseed cotyledons and the respective metabolomic and transcriptomic changes is missing. Such data are highly significance for the development of rapeseed genotypes that will be able to survive cold stress during the germination stage when cotyledons are being developed and seedlings are transitioning from the heterotrophic to autotrophic stage.

The mechanisms through which plants sense, transmit signals, and tolerate cold include changes in cytosolic calcium levels, signal amplification through phospholipids, ROS homeostasis, and the activation of cold stress responsive genes/pathways [14]. Typical cold tolerance responses include the activation of amino sugar metabolism, nucleotide sugar metabolism, aspartate and glutamate metabolism, plant hormone signaling, flavonoid biosynthesis, and starch and sucrose metabolism [15]. Under cold stress, plants undergo osmotic adjustment. To do so, plants biosynthesize an array of osmolytes such as sugars, amino acids, polyamines, and polyols. Their biosynthesis is triggered by several signaling pathways, including mitogen associated protein kinase (MAPK) signaling, phytohormone signaling, and ROS signaling [16]. A key cold stress regulatory pathway is the ICE-CBF-COR pathway (ICE: inducer of CBF, CBF: C-repeat binding factor, COR: cold regulated genes) [17]. Apart from the ICE-CBF-COR pathway, phytohormones such as abscisic acid (ABA), auxins, cytokinins, and melatonin play roles in cold stress tolerance [18,19]. An understanding of differential changes in these pathways in cotyledons of rapeseed genotypes with contrasting cold sensitivity will be provide useful data. The period from emergence to seedling transition is critical for the survival of the rapeseed plant as cotyledons expand and provide food for the growing plant. Any injury or stress to the cotyledons can result in reduced yield and may compromise the survival of the seedling. For example, damage to cotyledons significantly reduces seed number through final plant size in *Medicago lupulina* [20]. Developments in sequencing technologies have also enabled characterization of transcripts and accurate quantification of the expression of RNA in plants [21]. Parallel developments in metabolomics have significantly improved our understanding of the changes in the metabolic pathways in plants under stress scenarios [22].

Here, we studied the physiological changes, including in electrical conductivity, MDA content, chlorophyll content, and fructose and glucose contents, in cotyledons of rapeseed genotypes with different cold stress sensitivities. We employed transmission electron microscopy, physiological analyses, metabolome profiling, full-length transcriptome sequencing using the Oxford Nanopore Technologies approach, and qRT-PCR analysis to study the cold stress responses in the two rapeseed genotypes.

## 2. Materials and Methods

### 2.1. Plant Material and Cold Treatment

Two rapeseed (*Brassica napus* L.) genotypes were sourced from the Key Laboratory of Crop Epigenetics Regulation and Development in Hunan Province, Hunan Agricultural University, Changsha, Hunan, China. XY15 is a conventional rapeseed variety, while GX74 was developed by ethyl methylsulfonate mutagenesis of the former variety. The seeds of GX74 used in this study were obtained after seven successive rounds of self-breeding [12]. Thirty seeds of each variety were soaked after surface-sterilization and were evenly planted in each pot (model: 2 gallons, top diameter 22.5 cm, bottom diameter 18.5 cm, height 20.5 cm) containing sterilized peatmoss. The temperature of the growth room was 20–22 °C and the dark/light cycle was 8 h/16 h. The light source was LED lights with the intensity of ~300 µmol/m^2^/s and 900 µmol/m^2^/s at the bench height and 10 cm below the LED bars, respectively. The seeds were allowed to grow for eight days until the cotyledons were fully unfolded. The grown seedlings of each genotype were divided into two groups, i.e., control (CK) and those treated with cold stress (LW) for each genotype; three pots for each variety and each treatment. The LW groups were placed in 0 °C for 18 h, whereas the CK groups were kept at 20–22 °C. The samples were collected for CK and LW, and were frozen with liquid nitrogen and stored at −80 °C.

### 2.2. Transmission Electron Microscopy

Fully unfolded cotyledons were taken as samples from CK and LW treatments. The samples were cut into 6 mm discs and pre-fixed with 3% glutaraldehyde and then fixed with 1% osmium tetroxide. The samples were dehydrated in a step-by-step fashion using gradients of acetone (30% → 50% → 70% → 80% → 90% → 95% → 100%). The dehydration step with the 100% concentration was repeated thrice, followed by infiltration and embedding. Then we prepared ultra-thin slices (60–90 nm), followed by spreading and scooping into copper grids. They were then stained with uranyl acetate for 10–15 min, followed by staining with lead citrate for 1 to 2 min at room temperature, and observed under JEM-1400FLASH transmission electron microscope (Japan Electronics Co., Ltd., Tokyo, Japan).

### 2.3. Phenotypic and Physiological Parameter Analyses

The cotyledons stored at −80 °C were used for physiological parameters. The cotyledons were processed for each determination test according to the respective kit’s manufacturer’s instruction. The fructose and glucose contents were determined using a Plant Tissue Fructose Content Assay Kit (Beijing Solarbio Science and Technology Co., Ltd., Beijing, China) and Glucose Content Assay Kit (Beijing Solarbio Science and Technology Co., Ltd., Beijing, China), respectively. The MDA content was determined using a Malondialdehyde Assay Kit (Jiancheng Bioengineering Institute, Nanjing, China) following the thiobarbituric acid method. The H_2_O_2_, pyruvic acid, and proline contents were measured using a Hydrogen Peroxide Assay Kit, Pyruvate Assay Kit, and Proline Assay Kit (developed by Jiancheng Bioengineering Institute, Nanjing, China), respectively, through the calorimetric method. The relative electrical conductivity (REC) was determined according to a method previously described by Bajji et al. [23], with minor modifications. All analyses were performed in three independent repeats (XYCK1-3, XYLW1-3, GX74CK1-3, and GX74LW1-3).

### 2.4. Metabolome Analysis

Twelve cotyledon samples were individually weighed (3 g each), crushed into powder, and freeze-dried in vacuum. To the individual samples (100 mg), chilled (−20 °C) 600 µL methanol (containing 4 ppm of 2-chloro-Lphenylalanine) was added in a 2 mL centrifuge tube and vortexed for 30 s. The samples were extracted overnight at 4 °C followed by centrifugation at 1188.24× *g* at 4 °C for 10 min. Prior to extraction, equal volumes of each of the samples in the batch were mixed and extracted, as for other samples. The supernatants were taken, filtered through a 0.22 µm polytetrafluoroethylene membrane (ANPEL Laboratory Technologies, Shanghai, Inc., Shanghai, China), and used for LC-MS detection. The chromatographic conditions for the analysis were as follows. We used a Thermo Vanquish (Thermo Fisher Scientific, Waltham, MA, USA) ultra-high performance liquid phase system with an ACQUITY UPLC^®^ HSST3 (2.1 × 150 mm, 1.8 µm) (Waters, Milford, MA, USA) column. The flow rate, column temperature, and injection volume were 0.25 mL/min, 40 °C, and 2 μL, respectively. For the positive ion mode, the mobile phase included formic acid acetonitrile (0.1%, C) and formic acid water (0.1%, D). The gradient elution program was set to following conditions: 0~1 min, 2% C; 1~9 min, 2%~50% C; 9~12 min, 50%~98% C; 12~13.5 min, 98% C; 13.5~14 min, 98%~2% C; 14~20 min, 2% C. For the negative ion mode, the mobile phase included acetonitrile (A) and 5 mM ammonium formate water (B); the gradient elution program settings were as follows: 0~1 min, 2% A; 1~9 min, 2%~50% A; 9~12 min, 50%~98% A; 12~13.5 min, 98% A; 13.5~14 min, 98%~2% A; 14~17 min, 2% A.

For the mass spectrometry, we used a Thermo Q Exactive mass spectrometer detector (Thermo Fisher Scientific, Waltham, MA, USA), electrospray ionization source (ESI), and positive and negative ion modes. The positive and negative ion spray voltages were 3.50 kV and −2.50 kV. The sheath and auxiliary gasses were 30 arb and 10 arb, respectively. The capillary temperature was set at 325 °C. The first-level full scan was performed with a resolution of 70,000, with an ion scanning range of *m*/*z* 81~1000, and the second-level cracking was performed by HCD such that the collision voltage was 30%, and the second-level resolution was 17,500. Ten ions were fragmented while dynamic exclusion was used to remove unnecessary MS/MS information.

### 2.5. Transcriptome Sequencing and Analyses

Total RNAs were extracted from the 12 cotyledon samples (3 cold-treated and 3 CK samples for each of the XY15 and GX74) using TRIzol^®^ Regent (Tiangen Biotech, Beijing, China) according to the manufacturer’s instructions. RNA degradation and contamination were checked via 1% agarose electrophoresis. RNA purity was assessed using a Nanodrop Spectrophotometer (SpectraMax^®^ QuickDrop^TM^ Molecular Devices, Shanghai, China). RNA integrity was determined using an Agilent 2100 Bioanalyzer (Agilent Technologies, Santa Clara, CA, USA), and RNA samples with RNA integrity numbers in the range of 8–10 were used for further downstream analysis. Next, we used oligo DT as a primer to reverse transcribe the mRNAs followed by low-cycle PCR amplification of full-length cDNAs. AMPure magnetic beads were used for purification, followed by addition of sequencing adapters. The libraries were sequenced on a sequencing platform of Oxford Nanopore Technologies via FLO-PRO002 chip (MetWare Biotechnology Co., Ltd., Wuhan, China).

In order to obtain only high-quality reads for further analysis, the raw ONT reads were filtered; the reads with a quality value <7 and those less than 50 bp in length were filtered out using NanoFilt (version 2.8.0) [24]. Next, we used SeqKit (version: 0.12.0) [25] with default parameters to obtain statistics of the sequencing data volume. The filtered full-length sequence was aligned with the reference genome according to minimap2 (version 2.17-r941) using SAMtools [26]. Pinfish (version 0.1.0) was used to construct a non-redundant set of transcripts. In the obtained redundant set, the sequences were aligned to the reference genome using StringTie (version 2.1.4). New transcripts and new genes were identified using gffcompare (version 0.12.1). TransDecoder was used to predict the coding sequence of the newly identified transcripts. The new transcripts were then annotated in Nr [27], Pfam [28], UniProt [29], KEGG [30], GO [31], and KOG/COG [32].

### 2.6. qRT-PCR Analyses

To ascertain the reliability and accuracy of the RNA-seq data, 15 genes were selected from prominent pathways/GO terms for validation by qRT-PCR. Total RNAs were extracted from the CK and LW of the two genotypes (XY15 and GX74) according to the RNA extraction procedure outlined above. cDNA was synthesized by a PrimeScript^TM^ 1st strand cDNA Synthesis Kit (Solarbio Science and Technology Co., Ltd., Beijing China) following the manufacturer’s instructions. The cDNA was diluted and amplified using a Light Cycl^®^ 96 Real Time PCR system (Roche Life Science, Shanghai, China) and SYBR^®^ Premix Ex Taq^TM^ kit (TaKara Biomedical Technology, Dalian, China) following the manufacturer’s instructions. The reaction conditions were 94 °C for 5 min, followed by 40 cycles of 94 °C for 15 s and 60 °C for 1 min. The *actin2* (*ACT2*) gene was used as an endogenous control [33]. The specific primers (Table 1) were designed with Primer software (v.6.24, Primer, Quebec City, QC, Canada). qRT-PCR analyses included three biological replicates for each CK and LW samples of XY15 and GX74.

### 2.7. Statistical Analyses

The physiological data collected were subjected to Student’s *t*-test at a *p*-value ≤ 0.05 using Microsoft Excel 2019^®^ (www.microsoft.com).

For metabolome data, Pearson’s correlation coefficient (PCC), principal component analysis (PCA), and cluster heatmap analysis were performed in R (https://r-project.org/). The differential metabolites were screened based on the criteria of |log2fc| > 0.26, pvalue (*t*-test) ≤ 0.1, and VIP(OPLS-DA) ≥ 0. The differentially accumulated metabolites (DAMs) were annotated in the KEGG database (Kyoto Encyclopedia of Genes and Genomes) and enriched and mapped to KEGG pathways [34].

For transcriptome data, the expression was quantified as transcripts per kilobase of exon model million mapped reads (TPM) using salmon (version 1.4.0). Read count was used to perform differential expression in DESeq 2 [35]. The abundance difference in each genotype (CKvsLW) were calculated based on the ratio of the TPM values and false discovery rate (FDR). The TPM of detected genes were used to conduct Pearson correlation and principal components analysis in R with corrplot and ggbiplot packages, respectively.

The DESeq package in R [36] was used to standardize the counts of each sample gene (thus using the base mean value to estimate the expression based on TPM, and using the negative binomial distribution test) to assess the significant difference between the read numbers. Genes/transcripts were considered to be differentially expressed when the value of log2 fold change was >1 or <−1 with FDR value ≤ 0.05 between the CK and LW groups in or between each genotype. The GO and KEGG pathway enrichment analyses of the differentially expressed genes (DEGs) were conducted using the hypergeometric test by comparison with the whole genome background. GO terms and KEGG pathways with FDR-corrected *p*-value ≤ 0.05 were considered as significantly enriched. Heatmaps of key DEGs were produced with values of TPM under CK and LW in each genotype using the pheatmap package in R [37].

For qRT-PCR data, the relative level of gene expression was computed using the 2^∆∆Ct^ formula [38].

## 3. Results

### 3.1. Transmission Electron Microscopy-Based Ultrastructure of XY15 and GX74 in Response to Cold Stress

**Figure 1:**
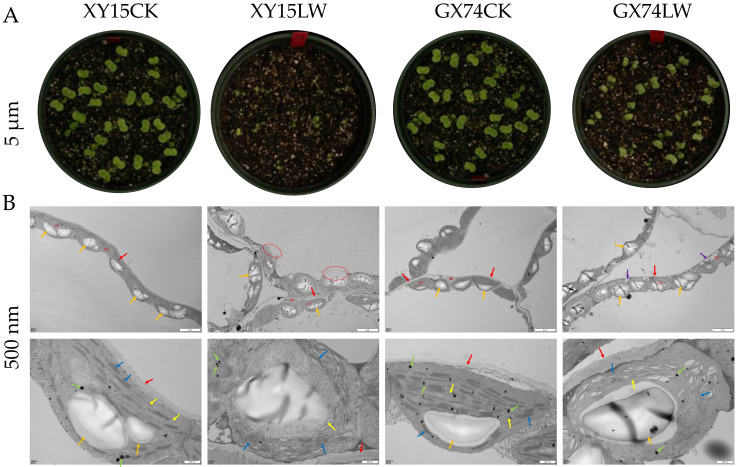
(**A**) Visual comparison of injury to the cotyledons of cold-sensitive (XY15) and -tolerant (GX74) *B. rapa* in response to cold stress (LW) compared to control (CK). (**B**) Transmission electron microscopic observations of mesophyll cells of XY15 and GX74 before and after cold stress treatment. Mi = mitochondria, CP = chloroplast, red arrow = cell wall, orange arrow = starch grains, green arrow = osmiophilic granules, blue arrow = crenellate, purple arrow = vacuole, and yellow arrow = lamellar. The red circles show that the outer membrane of the mesophyll cells is dissolved/ruptured.

The cotyledons of GX74 exhibited lower injury as compared to those of XY15 in response to the cold stress (Figure 1A). TEM revealed that the morphology of mesophyll cells in XY15CK and GX74CK cotyledons was normal with abundant cytoplasm, and the structure of each organelle could be visualized clearly. The chloroplasts were arranged neatly (Figure 1B). In contrast, in the case of XY15LW, the morphological structure of the mesophyll cells was severely damaged, and the cell membranes were discontinuous. The cytoplasmic content was diffuse due to dissolution and rupturing of the membrane (Figure 1B). In the case of GX74LW, the morphological structure of the mesophyll cells was slightly damaged with abundant cytoplasm. GX74LW had a small number of vacuoles and the chloroplasts were neatly arranged. Most of the chloroplasts had loose lamellar and stacked structures (Figure 1B). These observations indicate that the cotyledons of GX74 were least damaged compared to XY15 due to cold treatment. These results suggest that the former is a cold-tolerant genotype, whereas the latter is a cold-sensitive genotype.

### 3.2. Physiological Changes in Rapeseed Cotyledons (XY15 and GX74) in Response to Cold Stress

**Figure 2:**
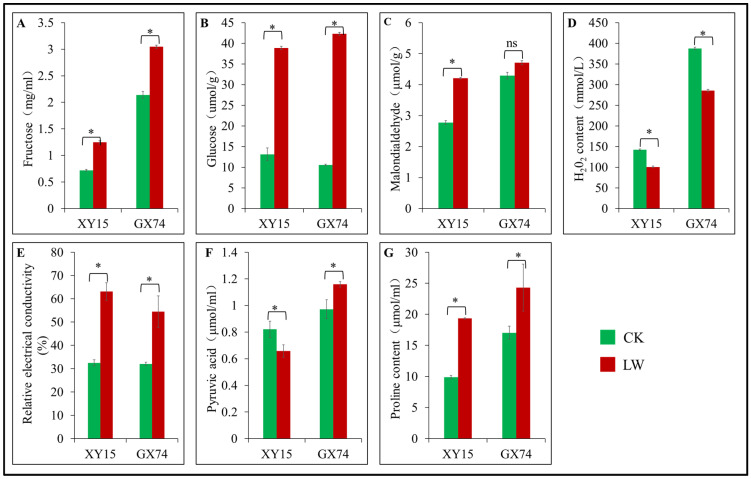
Physiological performance of cold-tolerant (GX74) and -sensitive (XY15) genotypes of *B. rapa* cotyledons before (LW) and after (18 h) of cold stress (LW). (**A**). Fructose content. (**B**). Glucose content. (**C**). Malondialdehyde content. (**D**). Hydrogen peroxide (H_2_O_2_) content. (**E**). Relative electrical conductivity. (**F**). Pyruvic acid content. (**G**). Proline content. Bars of a genotype with * and ns indicate significant and non-significant difference, respectively, between CK and LW. Error bars represent standard error of means of three replicates.

The physiological response data indicated that cold stress resulted in significantly (*p* < 0.05) higher accumulation of fructose, glucose, MDA, REC, and proline contents in both genotypes relative to their CKs (Figure 2A–E,G). However, the relative increase in REC was lower in GX74 compared to XY15, which is also consistent with the observed damage in mesophyll cells of the two genotypes (Figure 1). The MDA and proline contents of GX74 under CK and LW did not differ significantly (*p* > 0.05). Between the two genotypes, GX74 had higher contents of fructose, glucose, MDA, and proline. GX74 had 196.34% and 300.30% higher glucose contents than XY15 before and after cold stress, respectively. Cold stress significantly reduced H_2_O_2_ levels by 29.31% and 26.40% in XY15 and GX74 genotypes, respectively (Figure 2D). In addition, XY15 had a 19.81% reduction in pyruvic acid content in response to cold stress, while GX74 had a 19.23% increase in pyruvic acid content in response to cold stress (Figure 2F), suggesting that GX74 could better maintain the cell membrane stability compared with XY15. Since pyruvic acid connects glycolysis with the TCA cycle (KO00010 and KO00020) [39], its higher availability ensures better adaptation to cold stress. Most importantly, the TCA cycle acts as a bridge by connecting carbohydrates, lipids, proteins, and amino acid metabolism for maintenance of several processes, as well as to provide energy for metabolism [40,41]. Taken together, these physiological indicators show that cold stress significantly alters the physiology of the cotyledons of the two genotypes such that GX74 performs better than XY15.

### 3.3. Metabolome Profiles of XY15 and GX74 Challenged with Cold Stress

**Figure 3:**
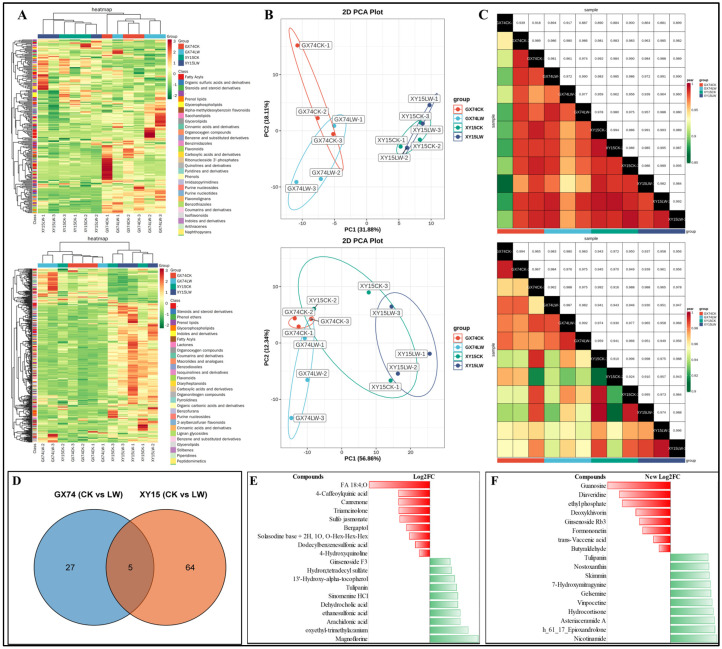
Metabolome analysis of *B. rapa* (XY15 and GX74) cotyledons challenged with cold stress. (**A**) Hierarchical heatmap clusters, (**B**) principal component analysis, and (**C**) Pearson’s correlation coefficient analyses of the detected metabolites in negative (upper panel) and positive modes (lower panel). (**D**–**F**) Differential metabolome analysis of *B. rapa* (XY15 and GX74) cotyledons in response to cold stress. (**A**) Venn diagram showing differential metabolites in GX74 (CKvsLW) and XY15 (CKvsLW). Log2 fold change of the highly up- and down-accumulated metabolites in (**B**) GX74 (CKvsLW) and (**C**) XY15 (CKvsLW).

The global metabolome profiling identified 590 compounds (220 in negative and 370 in positive ion mode). These metabolites belonged to 10 compound classes (Figure 3A). The PCA plots for both the negative and positive ion modes showed that the individual varieties tended to group together for CK and LW (Figure 3B). The PCC was higher than 0.85 for negative and 0.9 for positive ion modes (Figure 3C). These results indicate the reliability of the replicates and resulting datasets.

The differential analysis based on the screening criteria identified 32 and 74 DAMs in GX74 (CKvsLW) and XY15 (CKvsLW), respectively. Five DAMs were common between the two genotypes, namely, phenol lipid (3-Hydroxy-3′,4′-didehydro-beta,psi-caroten-4-one), fatty acyl (avocadene 2-acetate), amine (hexa(methoxymethyl)melamine), flavonoid (tulipanin), and an unclassified compound PE 36:3 (Table S1; Figure 3D).

In the case of GX74, 23 of the 32 DAMs were accumulated in higher quantities in LW compared to CK. The highly up-accumulated metabolites included magnoflorine, arachidonic acid, dehydrocholic acid, sinomenine HCl, tulipanin, coumaric acid 4-O-glucoside, primuline, and N-butylbenzenesulfonamide. In contrast, the compounds that showed reduced accumulation in response to cold stress in GX74LW included 4-O-caffeoylquinic acid (4-caffeoylquinic acid), canrenone, traiamcinolone, sulfo jasmonate, bergaptol, solasodine base+2H,1-O,O-Hex-Hex-Hex, dodecylbenzenesulfonic acid, and 4-hydroxyquinoline. Among these, arachidonic acid was enriched in multiple pathways including arachidonic acid metabolism, linoleic acid metabolism, secondary metabolite biosynthesis, unsaturated fatty acid biosynthesis, and metabolic pathways. Bergaptol and tulipanin were enriched in the biosynthesis of secondary metabolites and the anthocyanin biosynthesis pathway, respectively (Table S1; Figure 3E).

In the case of XY15, 59 of 74 DAMs were up-accumulated in response to cold stress. The highly up-accumulated metabolites included nicotinamide, h_61_17_epioxandrolone, asteriaceramide A, hydrocortisone, vinpocetine, gelsemine, 7-hydroxymitragynine, skimmin, nostoxanthin, and tulipanin. The DAMs that showed reduced accumulation in XY15LW compared to XY15CK include guanosine, diaveridine, deoxykhivorin, ginsenoside Rb3, formononetin, trans-vaccenic acid, and butyraldehyde. Among these, formononetin was enriched in metabolic pathways and biosynthesis of secondary metabolites. Others were enriched in ubiquinone and other terpenoid-quinone biosynthesis (gamma-tocotrienol), butanonate metabolism (butyraldehyde), nicotinante and nicotinamide metabolism (nicotinamide), carotenoid biosynthesis (nostoxanthin), anthocyanin biosynthesis (tulipanin), flavone and flavonol biosynthesis (a stragalin), metabolic pathways (hydrocortisone), biosynthesis of secondary metabolites (kaempferol 3-O-glucoside), and biosynthesis of cofactors (nicotinamide). Hydrocortisone is mostly detected in animal systems; therefore, its detection and role in plants should be further studied (Table S1; Figure 3E).

### 3.4. Full-Length Transcriptome Sequencing of XY15 and GX74 Challenged with Cold Stress

#### 3.4.1. Summary of Transcriptome Sequencing

**Figure 4:**
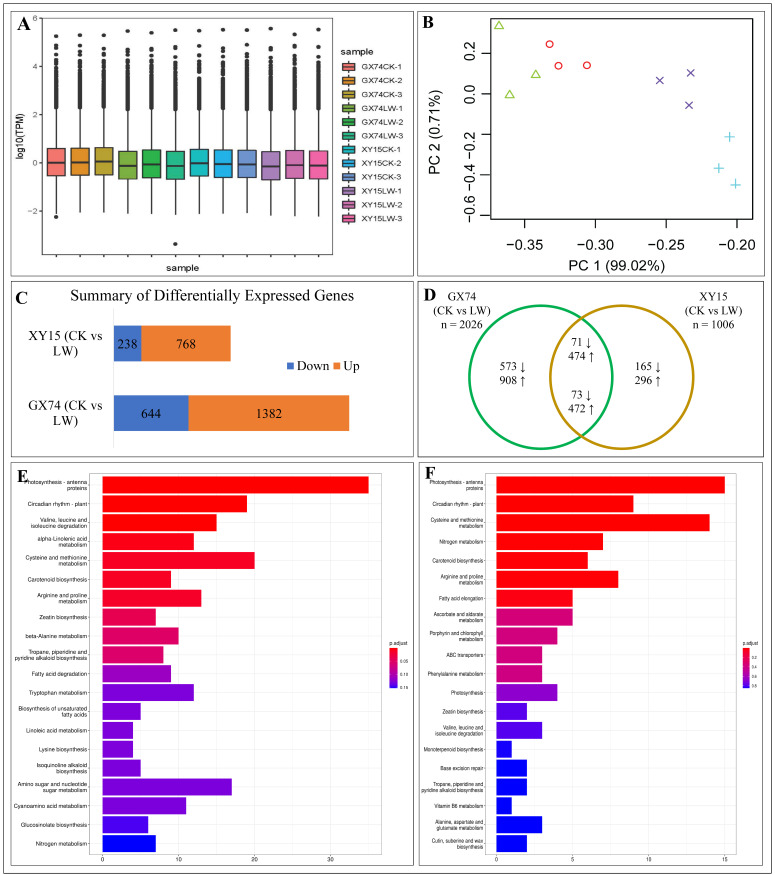
Overview of gene expression in *B. rapa* cotyledons in response to cold stress. (**A**) Distribution of average TPM and (**B**) principal component analysis in GX74 and XY15 before (CK) and after cold stress (LW). The numbers (1–3) with treatments indicate replicates. (**C**) Summary of differentially expressed genes and (**D**) Venn diagram of differentially expressed genes in GX74 and XY15 before and after cold stress. The up and down arrows indicate the number of up- or downregulated genes in cold treated samples compared to CK. KEGG pathway (top 20) enrichment barplots of DEGs in (**E**) GX74 (CKvsLW) and (**F**) XY15 (CKvsLW). *x*- and *y*-axis in (**E**,**F**) represent no. of DEGs and KEGG pathways. The color bars represent *p*-value (adjusted). The lower the *p*-value, the more significant the enrichment results.

Sequencing of 12 cDNA libraries produced 97,239,972 reads with an average of 8,103,331 per sample (Table S2). The average number of clean reads recorded in XY15CK, XYLW, GX74CK, and GX74LW was 7,462,187, 7,968,950, 7,120,774, and 7,911,979, respectively. The full-length read % was 84.30, 84.82, 84.86, and 84.27% for XY15CK, XYLW, GX74CK, and GX74LW, respectively (Table S2). A total of ~88.23% clean reads could be mapped to the reference genome “Darmor-bzh” [42], whereas 103,722 transcripts (including 101,040 known and 2682 novel transcripts) were obtained from the 12 libraries. These novel transcripts were identified from the consensus set of transcripts. On average, there were 20,085 and 17,214 consensus transcripts in GX75 CK and LW, respectively. The number of average consensuses set of transcripts in XY15 CK and LW was 17,674 and 17,271, respectively. The maximum and mean lengths of the transcripts were 12,677 and 665 bp, respectively, and the mean N50 length was 873 bp (Table S2). These transcripts were annotated as 102,759 genes. This significantly higher number of genes/transcripts also suggests detection of homeologs [43] as *B. napus* is a polyploid species. The gene expression (TPM) distribution in the twelve samples is shown in Figure 4A. The first two axes of PCA exhibited 99.73% variability among the samples (Figure 4B).

#### 3.4.2. Comparative Expression Profiles of XY15 and GX74 Cotyledons Challenged with Cold Stress

A total of 2487 DEGs were expressed in the two genotypes based on the screening criteria of log2FC ≥ 1 and FDR with the adjusted *p*-value (padj) < 0.05 [44]. Of these, 1006 DEGs (238 downregulated and 768 upregulated) and 2026 DEGs (644 vs. 1382) were identified in XY15 (CKvsLW) and GX74 (CKvsLW), respectively (Figure 4C). The lower number of DEGs in GX74 is consistent with that of the DAM results (Table S1; Figure 4). Generally, the relatively higher numbers of upregulated genes and DAMs indicate that cold stress alters the expression and accumulation of a large number of genes and metabolites, respectively.

The comparative analysis identified that 461 and 1481 DEGs were exclusive to XY15 and GX74, respectively (Figure 4C; Table S3A,B), whereas 545 DEGs were common between the two genotypes (Figure 4D; Table S3C). These could be involved in plant growth and development, whereas those expressed exclusively in each genotype are potentially related to cold stress tolerance/susceptibility.

The DEGs in XY15 (CKvsLW) were significantly enriched in a number of KEGG pathways including photosynthesis-antenna proteins, circadian rhythm–plant, cysteine and methionine metabolism, nitrogen metabolism, carotenoid biosynthesis, arginine and proline metabolism, and fatty acid elongation (Figure 4E). The DEGs in GX74 (CKvsLW) were significantly enriched in photosynthesis-antenna proteins, carotenoid biosynthesis, circadian rhythm–plant, amino sugar and nucleotide sugar metabolism, and several metabolic pathways (Figure 4F). Consistently, the GO enrichment analysis showed that the DEGs were mainly enriched in photosynthesis- and chlorophyll-related terms (GO:0009765, GO:0016168, GO:0009523, GO:0009768, and GO:0009522), rhythmic process/circadian rhythm (GO:0048511, GO:0007623, and GO:0042752), cell-related terms (GO:0009535, GO:0009664, GO:0005615, GO:0008360, GO:0042546, and GO:0008283), response to phytohormones (GO:0009733 and GO:0009737), response to ROS-related terms (GO:0042542 and GO:0004096), and several other terms (Figure S1A,B). Consistent with earlier studies on the role of antenna proteins [45], circadian rhythm [46], and other pathways, these results suggest that both genotypes activate a number pathways in modulating their response to cold stress.

In the case of XY15, we found that GATA TF, glycol_hydro_19_cat domain-containing protein, RING-type E3 ubiquitin transferase, TPR_region domain-containing protein, and xyloglucan endotransglucosylase/hydrolase (XTH) genes exhibited reduced expression upon cold stress (Figure S2A). In contrast, genes including AP2/ERF domain-containing protein, CCT domain-containing protein, RING-type E3 ubiquitin transferases, RRM domain-containing protein, and several uncharacterized genes showed increased expressions in XY15LW as compared to XY15CK (Figure S2B). In the case of GX74, DEGs including SHSP domain-containing protein, AP2/ERF domain-containing proteins, XTHs, extensin-3 protein, bZIP domain-containing protein, and several uncharacterized genes were downregulated after cold stress (Figure S2C). On the contrary, the expressions of AP2/ERF domain-containing proteins, HTH La-type RNA-binding domain-containing protein, RING-type E3 ubiquitin transferase, RRM domain-containing protein, and S-acyltransferase (Figure S2D) increased upon cold stress in GX74. Interestingly, half of these genes did not express in CK; thus, they are specific to cold stress. Taken together, these observations indicate that cold stress tolerance significantly alters the cell wall, s-acylation, ubiquitin proteasome pathway, and plant hormone signaling.

##### A—Expression Changes in Genes Related to Physiological Changes

**Figure 5:**
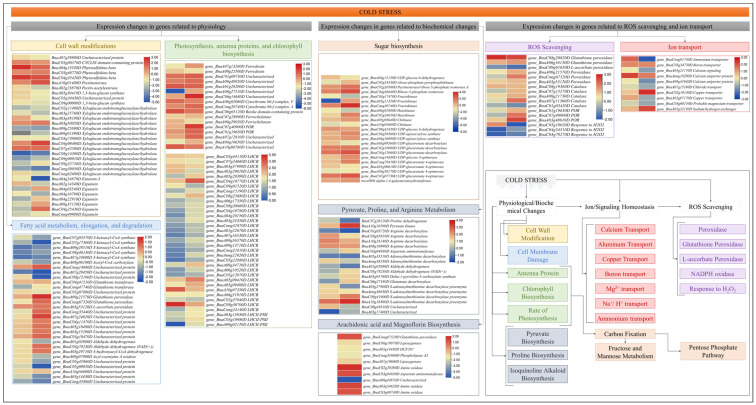
Effects of cold stress on gene expression related to physiological and biochemical changes, ion homeostasis, and ROS scavenging. The heatmaps are based on the log2 fold change values. The left and right columns of the heatmap represent XY15 (CKvsLW) and GX74 (CKvsLW), respectively. The colors of the boxes in the pathway correspond to the borders of the heatmaps.

When freezing-induced dehydration exceeds the tolerance levels of the cells, it affects the cells walls and damages cell membranes [47]. In this regard, we mined for DEGs related to cell and water deprivation/desiccation/dehydration-related GO terms i.e., cell wall biogenesis (GO:0042546), extracellular space (GO:0005615), cellular amino acid biosynthetic process (GO:0008652), cell wall organization (GO:0071555), cell population proliferation (GO:0008283), and regulation of cell shape (GO:0008360). In all, 33 DEGs were identified; 14 were expressed in XY15 (LWvsCK) and 26 in GX74 (LWvsCK) (Figure 5). In the case of XY15, only a pectinesterase (*BnaA10g01400D*), two phytosulfokine-betas (*BnaC04g35420D* and *BnaA04g13330D*), and one CYCLIN domain-containing protein (*BnaC03g00570D*) were upregulated in response to cold stress, whereas other DEGs including XTHs and expansins were downregulated. In the case of GX74, cold stress resulted in up- and downregulation of 8 and 18 genes, respectively, as compared to CK. The upregulated genes include expansin (*BnaC04g25430D*), three XTHs (*BnaA03g50050D*, *BnaC04g08400D*, and *BnaC07g42500D*), a pectinesterase (*BnaA10g01400D*), and three phytosulfokine-betas (PSKβ, *BnaA04g13330D*, *BnaC04g35420D*, and *BnaA10g01400D*), whereas the downregulated genes included a pectin acetyl esterase, two 1,3-beta-glucan synthases, three expansins, and 10 XTHs. These expression changes indicate that cold stress affects the cell wall-related genes’ expression and most are downregulated. However, the increase in the expressions of one expansin, three PSKβs, and three XTHs could be a possible reason for better cold stress tolerance or per se less injury to the cell in GX74 as compared to XY15.

In addition to the cell wall, cold stress also affects the cell membrane. The significant increase in MDA content in XY15 and non-significant changes in GX74 after cold stress indicate that the former genotype experiences a higher rate of oxidation of membrane lipids (Figure 2C). Consistently, we also observed increased accumulation of compounds classified as lipids and lipid-like molecules; there was a larger number of up-accumulated lipid compounds in XY15 compared to GX74 (Table S1). In this regard, we observed that a relatively higher number of genes involved in fatty acid elongation (ko00062) was repressed in GX74 (*BnaC07g05570D*, *BnaC07g17300D*, *BnaA09g28510D*, *BnaC08g46130D*, and *BnaA07g24600D*) compared to XY15 (*BnaA09g28510D*, *BnaC08g46130D*, and *BnaA07g24600D*) in XY15. Furthermore, an acetyl-CoA carboxylase gene (*BnaA08g06180D*) involved in fatty acid metabolism (ko01212), a glutathione S-transferase (GST, *BnaA07g28450D*) enriched in glutathione metabolism (ko00480), an aldehyde dehydrogenase gene (*BnaA01g02880D*) enriched in fatty acid degradation (ko00071), and three other lipid metabolism (ko00565) genes (*BnaC05g09920D*, *BnaA05g14430D*, and *BnaCnng35860D*) were downregulated in GX74 in response to cold stress. Interestingly, these genes did not differentially express in XY15. On the other hand, fifteen DEGs, including five fatty acid metabolism genes, five glutathione metabolism genes, and five fatty acid degradation genes, were upregulated only in GX74 (Figure 5). These expression changes, together with the MDA content and accumulation of lipid and lipid-like compounds in both genotypes, indicate that GX74 experiences relatively less lipid peroxidation than XY15.

Cold stress also causes reduction and impairing of photosynthesis, antenna proteins, and chlorophyll biosynthesis [48]. A search for the genes enriched in photosynthesis (ko00195), photosynthesis-antenna proteins (ko00196), and porphyrin and chlorophyll metabolism (ko00860) resulted in the identification of 9, 35, and 8 genes, respectively. Overall, there was a higher number of DEGs in GX74 compared to XY15. Generally, their expressions were relatively higher in the former than the latter in CK as well as LW. In the case of photosynthesis, the expressions of cytochrome b6-f complex (cyt b6/f) and ferredoxin (Fd) genes were enhanced upon cold stress in both genotypes in response to cold stress. This is also consistent with the upregulation of NADPH-protochlorophyllide oxidoreductases (NADP-POR) in GX74 in cold stress. Moreover, 35 genes annotated as antenna proteins (light-harvesting chlorophyll a/b-binding, LHCB) were differentially expressed; 14 and 30 in XY15 (CKvsLW) and GX74 (CKvsLW), respectively. More of these were upregulated in response to cold stress in GX74 than in XY15. We also observed that chlorophyll a-b binding protein 1 and chloroplastic LHCII type I (LHCII) were upregulated in both genotypes in response to cold stress (Figure 5). The upregulation of a relatively higher number of genes in GX74 as compared to XY15 possibly enables the former to tolerate cold stress by stabilizing antenna proteins and enhancing or maintaining the photosynthesis rate.

##### B—Expression Changes in ROS Scavenging and Ion Homeostasis Related Genes

Excessive generation of ROS under cold stress causes severe oxidative damage. Plants mitigate these effects by activating the antioxidant defense mechanisms [40]. In this regard, we explored expression changes in ROS-scavenging-related genes, i.e., POD, glutathione peroxidase (GPx), L-ascorbate peroxidase (APx), and catalase (CAT). Two POD genes (*BnaC08g20820D* and *BnaA06g16150D*) were upregulated in response to cold stress in both genotypes with higher expressions in GX74 than XY15 under both CK and LW (Figure 5), whereas two GPx (*BnaCnng67520D* and *BnaA09g21570D*), one APX (*BnaA03g53120D*), one POD (L-ascorbate POD, *BnaC09g01650D*), and four CAT (*BnaC08g19360D*, *BnaC07g15270D*, *BnaA08g21730D*, and *BnaA07g11360D*) genes were upregulated in GX74 in response to cold stress but did not differentially express in XY15 (Figure 5). Among other oxidoreductases, i.e., nicotinamide adenine dinucleotide phosphate (NADPH) oxidase or Rboh [49], three NADPH-protochlorophyllide oxidoreductase (POR) encoded genes (*BnaC01g19630D*, *BnaC07g40900D*, and *BnaA03g48610D*) were exclusively upregulated in GX74 upon cold stress (Figure 5). We also found six genes involved in response to H_2_O_2_ (GO:0042542) (Figure 5), of which four genes (*BnaA07g11360D*, *BnaA08g21730D*, *BnaC07g15270D*, and *BnaC08g19360D*) were upregulated in GX74 upon cold stress, whereas only *BnaC04g54510D* and *BnaC03g78270D* were differentially expressed in XY15. Relatively higher TPM values of these genes in GX74 as compared to XY15 are consistent with the higher H_2_O_2_ production (Figure 2D). The higher expressions of POD, GPx, APx, and CAT genes in response to cold stress in GX74 possibly enabled relatively greater ROS scavenging.

We found seventeen DEGs related to ion transport/homeostasis in GX74 (CKvsLW). Contrastingly, only two genes were expressed differentially in XY15; a Ca^2+^ uniporter protein (*BnaAnng18280D*) was upregulated in both genotypes and one Al^3+^ transporter protein (*BnaCnng01740D*) was downregulated in GX74, but was upregulated in XY15. In the case of GX74, a Mg^2+^ protein (*BnaA05g21170D*), two Cu^2+^ proteins (*BnaC02g10130D* and *BnaA02g07170D*), two Ca^2+^ proteins (*BnaA07g12410D* and *BnaCnng26490D*), one Na^+^/H^+^ exchanger protein (NHX, *BnaA05g32130D*), and one boron transporter protein (*BnaC03g34730D*) were upregulated in response to cold stress. The protein membrane transporters maintain cellular homeostasis by controlling the ion transport across membranes during abiotic stresses. The results showing that several Mg^2+^, Cu^2+^, and Na^+^/H^+^ proteins were upregulated in GX74 after cold stress indicate that the genotype is able to maintain homeostasis better than XY15 [50]. However, six other Ca^2+^ proteins and one Mg^2+^ transporter protein were downregulated upon cold stress in GX74 (Figure 5). The expressions of these genes indicate that GX74 employs different ion transporters for homeostasis to tolerate cold stress, whereas XY15 does not involve such a large number of genes for homeostasis and, therefore, cannot tolerate cold stress.

##### C—Differential Expression of Genes Related to Biochemical Changes

i. Expression changes in sugar biosynthesis-related genes

**Figure 6:**
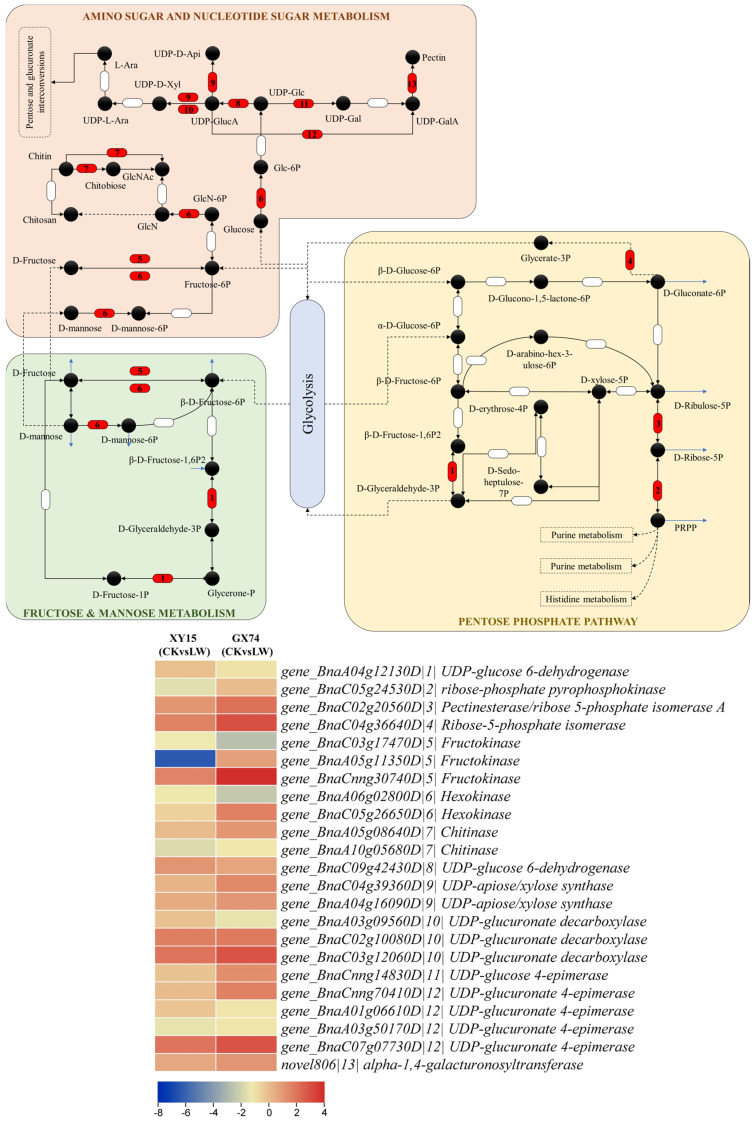
Expression changes in sugar biosynthesis-related KEGG pathways. The genes given as red semi-circles were differentially expressed in the *B. rapa* (XY15 and GX74) cotyledons before and after cold stress treatment. The heatmap on the right panel represents log2 fold change values of the DEGs enriched in the KEGG pathways; gene id is followed by a number (given as |x|) corresponding to the number given in the red semi-circle. The number is followed by gene annotation according to KEGG database.

The differential expression of the genes involved in porphyrin and chlorophyll metabolism (Figure 5) and the higher levels of fructose and glucose observed in GX74 compared to XY15 cotyledons (Figure 2) indicate that under cold stress conditions, GX74 cotyledons have higher sugar levels than those of XY15. In this regard, we observed the expression changes in genes enriched in sugar-related pathways i.e., pentose phosphate pathway, fructose and mannose metabolism, and amino sugar and nucleotide sugar metabolism. We observed that the gene related to D-fructose and β-D-fructose interconversion (*BnaA04g12130D*) showed reduced expression in LW compared to CK in both genotypes, whereas the gene controlling conversion of D-gluconate-6P to glycerate-3P (*BnaC04g36640D*) showed upregulation in both genotypes such that GX74 had higher expression than XY15. Two of the three fructokinases (*BnaA05g11350D* and *BnaCnng30740D*) were upregulated in GX74LW compared to CK. Interestingly, we observed upregulation of a hexokinase (HK, *BnaC05g26650D*) in GX74LW compared to CK, whereas the other HK (*BnaA06g02800D*) was downregulated. Similarly, the two chitinases showed contrasting expressions in GX74 upon cold stress treatment. Moving further downstream in the amino sugar and nucleotide sugar metabolism pathway, the genes involved in UPD-glucose biosynthesis, and its conversion to UDP-GlcA and then to UDP-D-xylose and UDP-D-apiose, were upregulated in GX74LW compared to CK. Furthermore, UDP-glucose 4-epimerase (*BnaCnng14830D*), which converts UDP-Glc to UDP-gal, was upregulated in GX74LW compared to CK. UPD-glucose is a key metabolite involved in several pathways including cell wall synthesis, remodeling, zeatin, secondary metabolites, cofactors, and nucleotide sugar biosynthesis (KEGG compound C00029). Hence, the upregulation of related genes is a possible reason explaining why GX74 can tolerate cold stress better than XY15 (Figure 5 and Figure 6). The upregulation of these genes in GX74 could be associated with the higher sugar contents measured in the cotyledons (Figure 2A,B). This in turn might trigger cold tolerance in GX74 [51].

ii. Expression changes in genes related to arachidonic acid and magnoflorine

Since the highest accumulated metabolites in GX74 after cold stress were magnoflorine and arachidonic acid (Table S1), we observed the expression changes in related genes. Regarding arachidonic acid, six genes enriched in arachidonic acid biosynthesis and/or linoleic acid metabolism pathways were differentially expressed. Two GSTs and an amine oxidase were upregulated, while two lipoxygenases and another amin oxide were downregulated in response to cold. Considering the positions of these genes in respective pathways (arachidonic acid biosynthesis and/or linoleic acid metabolism), it could be said that instead of linoleate conversion to 13S-hydroperoxy-9Z,11E-octadecadienoic acid, cold stress induces the expression of GSTs to increase arachidonic acid biosynthesis. In the case of magnoflorine, three amine oxidases and an aspartate aminotransferase were upregulated in the isoquinoline alkaloid biosynthesis pathway (Figure 5). These genes convert L-tyrosine to precursors used for magnoflorine biosynthesis; therefore, it is understandable that they might play a significant role in cold stress tolerance.

iii. Expression changes in genes related to pyruvate, arginine, and proline metabolism

We also explored DEGs to understand the basis for the difference in pyruvic acid contents observed in the cotyledons between the genotypes (Figure 2F). Two pyruvate metabolism (ko00620) genes, i.e., pyruvate kinases (PKs, *BnaC08g49310D* and *BnaA10g10590D*), were expressed in both genotypes with higher expressions in GX74LW than either XY15LW or XY15CK. Of these, only *BnaA10g10590D* was differentially expressed in GX74 in response to cold stress. Interestingly, its expression reduced in XY15 in response to cold stress, which is consistent with the lower pyruvic acid contents in XY15 as compared to GX74. In contrast, regarding proline content changes in response to cold stress, we found 15 DEGs enriched in arginine and proline metabolism (ko00330); eight in XY15 (LWvsCK) and 13 in GX74 (LWvsCK) (Figure 5). In the case of XY15, four genes (*BnaA03g27900D*, *BnaAnng36830D*, *BnaC09g41900D*, and *BnaA10g18360D*) encoding for S-adenosylmethionine decarboxylase proenzyme (SAMDC1) and another four genes were upregulated in response to cold stress. It is an important observation since S-adenosylmethionine decarboxylase is involved in biosynthesis of plant hormones (KEGG compound C00019). In GX74, nine and four DEGs were up- and downregulated in response to cold stress, respectively. From these, one proline dehydrogenase encoded gene (*BnaC07g26120D*) decreased in expression by 52.72% upon cold stress in GX74, even though this same gene did not express differentially in XY15, but the latter genotype had an increased expression (8.71%) upon cold stress. Of the SAMDC1 genes only, *BnaC09g41900D* and *BnaA10g18360D* were upregulated in GX74 in response to cold stress. The larger number of upregulated DEGs in GX74 may be responsible for the higher accumulation of proline in GX74 at both CK and LW than XY74 (Figure 2G), as it has been reported that overproduction of proline in plants regulates stress tolerance by maintaining cell turgor/osmotic balance, stabilizing membranes and thereby controlling electrolyte leakage, and bringing concentrations of ROS within normal ranges [52].

##### D—Expression Changes in Phytohormone Biosynthesis/Signaling and ICE-CBF-COR Pathway

**Figure 7:**
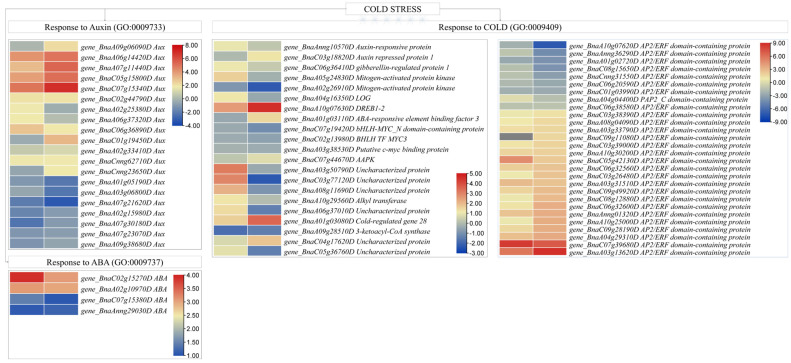
Expression changes in auxin, abscisic acid, and cold-responsive genes in *B. rapa* (XY15 and GX74) cotyledons in response to cold stress. The heatmaps represent log2 fold change values.

Twenty-five DEGs annotated as auxin-responsive genes (GO:0009733) could be screened between the two genotypes. Six and ten gens were upregulated in XY15 and GX74 cotyledons, respectively, in response to cold stress. Of these, *BnaA09g06090D* was exclusively expressed (upregulated 4.55-fold) in GX74 in response to cold stress. Contrarily, three (*BnaA01g05190D*, *BnaA03g06800D*, and *BnaA07g21620D*) and five (*BnaA07g21620D*, *BnaA02g15980D*, *BnaA07g30180D*, *BnaA07g23070D*, and *BnaA09g38680D*) AUX genes were downregulated in GX74 and XY15 after cold stress. Generally, the upregulated DEGs had higher TPM values in GX74 as compared to XY15. Apart from auxin, four ABA responsive genes (GO:0009737) (*BnaC02g15270D*, *BnaA02g10970D*, *BnaC07g15380D*, and *BnaAnng29030D*) were upregulated in both XY15 and GX74 cotyledons in response to cold stress; however, their TPM values were mostly higher in GX74 than XY15 (Figure 7). These expression trends indicate that under the influence of colds stress, GX74 activates a relatively higher number of genes associated with auxin responses compared to XY15.

Seventeen ICE-CBF-COR pathway-related DEGs were identified from KEGG and GO analyses (Figure 7; Tables S3 and S5). From these, five ICE related genes/proteins (Auxin-responsive protein (*BnaAnng10570D*), gibberellin-regulated protein 1 (*BnaC06g36410D*), mitogen-activated protein kinase (MAPK, BnaA05g24830D), and cytokinin riboside 5′-monophosphate phosphoribohydrolase (LOG, *BnaA04g16350D*)) were upregulated in XY15 in response to cold stress, whereas one auxin repressed protein 1 (*BnaC03g18820D*) and one MAPK (*BnaA02g26910D*) were up- and downregulated in GX74 in response to cold stress, respectively. One DREB1-2 gene (*BnaA10g07630D*) involved in CBF was upregulated in both XY15 and GX74, while one ABA-responsive element binding factor 3 (ABF3, *BnaA01g03110D*) was exclusively upregulated in GX74LW compared to GX74CK (Figure 7). Moreover, nine genes were identified for response to cold (GO:0009409) from the GO analysis. Six of the COR (GO:0009409)) genes (*BnaA03g50790D*, *BnaC03g77120D*, *BnaA08g11690D*, *BnaA10g29560D*, *BnaA06g37010D*, and *BnaA01g03080D*) were upregulated in XY15; however, only *BnaA01g03080D* was upregulated in GX74. The expressions of all of the above listed six COR genes were higher in GX74 upon cold stress (Figure 7). Since CBFs are APETALLA2 (AP2)-type transcriptional activators (TAs) and constitute a central component of cold signaling networks that elicit freezing tolerance [53], we searched for the expression changes in these genes. Twenty-eight and eleven AP2 gene family members were expressed differentially in GX74 and XY15, respectively (Figure 7). Of these, *BnaA10g07620D* showed zero expression upon cold stress in GX74, whereas *BnaC09g11080D* was exclusively upregulated in GX74. These expression changes suggest that the ICE-CBF-COR pathway is active in both genotypes; however, a relatively larger number of transcripts/genes are activated in cold stress in GX74, enabling it to survive under cold stress.

##### E—Expression Changes in Transcription Factors

A total of 706 DEGs were identified belonging to B3, bHLH, bZIP, C2H2, ERF, FAR1, MAD, MYB, NAC, and WRKY TF families (Table S4) with different levels of regulation in the two genotypes (Table 2). Notably, there were higher numbers of differentially expressed TFs in GX74 as compared to XY15, which could be a reason for the induction of the relatively higher number of DEGs in the former than the latter.

Out of 20 B3 TFs, only *BnaC01g39520D* was exclusively expressed in XY15 (Table S4), while more than half of the 19 B3 TFs were upregulated in GX74 in response to cold stress. Three bHLH TFs (*BnaC09g09850D*, *BnaCnng23400D*, and *BnaC03g60420D*) were downregulated, while two bHLH TFs (*BnaA05g21170D* and *BnaC04g35320D*) were upregulated in cold-treated GX74 cotyledons; all of these five bHLH TFs were exclusively expressed in GX74. (Table S4), whereas one bHLH TF (*BnaA05g29020D*) was only expressed after cold stress treatment in XY15LW.

Out of 38 bZIP genes, two (*BnaC01g17820D* and *BnaAnng09500D*) were upregulated in XY15 in response to cold stress. In the case of GXY4, two bZIP TFs, i.e., *BnaC02g06270D* and *BnaC01g01320D*, had contrasting expression trends. Earlier studies on brassica have revealed that bZIP TFs are involved in cold stress responses [54]. Fifty-nine C2H2 genes were detected, out of which only *BnaA03g17400D* was solely expressed (downregulated) in XY15 and the other two (*BnaC01g33270D* and *BnaC09g19820D*) were downregulated in GX74. C2H2 TFs play diverse roles in plants, including cold stress tolerance as well as hormone signal transduction, e.g., *AtZAT12* imparts tolerance against cold and oxidative stresses [55]. The majority of the TFs were classified as ERF TFs (156). Three of these (*BnaC02g36690D*, *BnaC09g11080D*, and *BnaA09g02120D*) showed increased expressions in response to cold stress in GX74. Furthermore, one ERF gene (*BnaA02g01020D*) had a 15.26-fold increase in expression upon cold stress in XY15, but was not expressed in GX74. ERFs are known for their roles in cold stress tolerance in plants [56]. Of the forty FAR1 TFs, three (*BnaA02g07880D*, *BnaCnng14830D,* and *BnaA09g01870D*) were exclusively expressed (upregulated) in cold-treated GX74 cotyledons. Among the 39 MAD genes identified, only two, i.e., *BnaAnng08150D* (downregulated) and *BnaC09g33710D* (upregulated), were expressed in GX74 (Table S4).

There were 125 differentially expressed MYB TFs; of these, 2 were downregulated (*BnaC07g27140D* and *BnaA09g01390D*) and 3 were upregulated (*BnaC06g05650D*, *BnaCnng38000D*, and *BnaA09g06090D*) in GX74 in response to cold stress. These five MYB TFs were exclusively expressed in GX74. MYB TFs increase cold hardiness by CBF-dependent (and -independent) pathways [57]. Thus, these results provide a list of important MYB TFs for further characterization in GX74 under a cold stress scenario. In contrast, of the 73 NAC TFs, only *BnaA09g02250D* was exclusively upregulated in GX74. Similarly, only *BnaA09g03940D* and *BnaC09g13550D* out of 40 WRKY genes were exclusively expressed (downregulated) in GX74 (Table S4). WRKYs act as molecular switches and regulate abiotic stress responses in plants [58,59].

The major TF families identified in this study highlight their involvement in modulating rapeseed cotyledons’ response to cold stress. The exclusively expressed TF genes in either of the genotypes, as well as those mutually detected in both genotypes, would be useful for functional validation experiments in deepening our understanding of their regulatory roles in the two genotypes in responding to cold stress.

**Figure 8:**
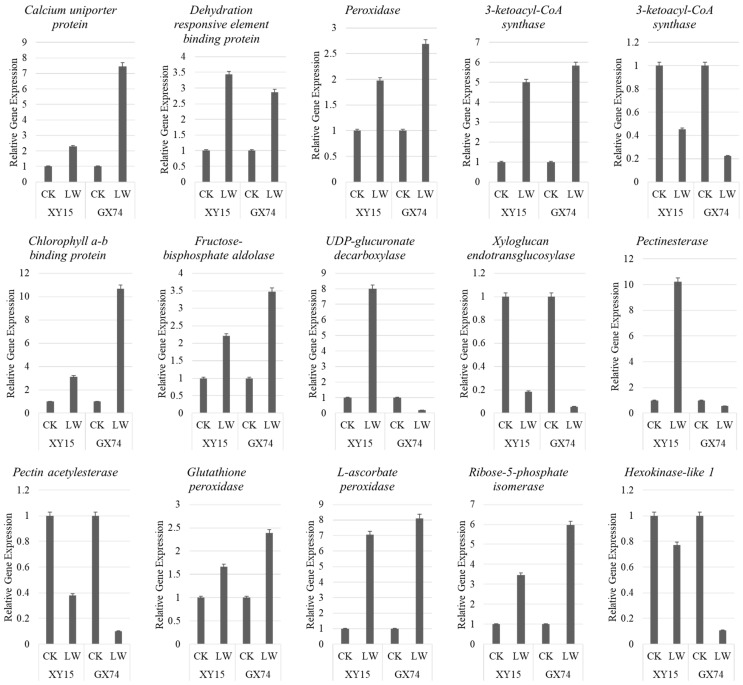
Quantitative real-time PCR analysis of *B. napus* genes in XY15 and GX74 before and after cold stress. The bars are means of three replicates. The error bars represent standard deviation.

To further validate the transcriptome sequencing results, we studied the expression of the 15 genes through qRT-PCR analysis. The results showed that the PCC (R^2^) was 0.83, indicating that transcriptome sequencing results are reliable (Figure 8; Figure S3).

## 4. Discussion

Cold stress is an important yield-limiting factor in brassicas as it affects different pathways in different periods of stress [60]. In our earlier work, we explored the metabolome and transcriptome profiles of siliques of XY15 and GX74 genotypes before and after cold stress [12], where we shed light on the key metabolomic responses and respective expression changes. However, apart from studying the effect of cold on siliques, it is still necessary to understand how low-temperature stress affects the early plant growth. It is important because >90% of rapeseed is winterized, and thus sown late, meaning seedlings are likely to experience low-temperature stress of 0–4 °C after germination. Such stress causes a yield reduction [2]. In this regard, here we report the key changes in ROS scavenging, sugar biosynthesis, and chlorophyll biosynthesis, and explore the differential expression by transcriptome sequencing in the cotyledons of GX74 and XY15.

### 4.1. Role of Sugar Biosynthesis in Contrasting Cold Stress Tolerance in Rapeseed Cotyledons

Sugars help in increasing the stability of phospholipidic mono- and bilayers. Studies have indicated that subcellular changes in these sugars are directly associated with the success of the responses to cold stress [51]. The higher fructose and glucose levels in GX74 as compared to XY15 both under CK and LW conditions (Figure 2) clearly indicate that GX74 has better potential to cope with cold stress responses. These observations are consistent with earlier reports that cold-stressed *Jatropha curcas* showed higher cotyledon fructose and glucose levels in response to cold stress [61]. The increase in fructose and glucose contents is also consistent with a higher number of DEGs in GX74 (LWvsCK) as compared to XY15 (LWvsCK) (Figure 4C). The increase in fructose content in response to cold stress in both genotypes can be associated with PfkB domain-containing protein and fructose-bisphosphate aldolase, because in Arabidopsis, the expression of fructose-bisphosphate (*AtFBA3* and *AtFBA4*) aldolase increased as compared to the wild type [62]. Furthermore, the ectopic expression of a coconut FBA gene in Arabidopsis resulted in an increased accumulation of substrates involved in sugar metabolism [63]. By comparison, the upregulation of a UDP-glucose 4-epimerase and four UGDHs in GX74 in response to cold stress indicates that UDP-Glc is being converted to UDP-GlcA as well as UDP-Gal (Figure 6), which is present upstream in the starch and glucose metabolism, as well as the pentose and glucuronate metabolism [64]. Thus, the resources in GX74 are directed toward higher fructose and glucose biosynthesis. An earlier study reported that overexpression of a UGDH from *Larix gmalinii* enhanced the growth and cold tolerance in transgenic Arabidopsis [65]. Moreover, the increased contents of fructose and glucose in GX74 in response to cold stress compared to XY15 may also be due to the higher expressions of cyt b6/f and Fd enriched in photosynthesis, and POR enriched in porphyrin (and chlorophyll) metabolism (Figure 5). Earlier studies have shown that POR overexpression increases chlorophyll biosynthesis, photosynthetic proteins, and pigments, and inhibits ROS production [66]. By comparison, the constitutive overexpression of ferredoxin-like protein in rice resulted in higher fructose and glucose levels in rice, together with increased photosynthesis [67]. Thus, GX74 has higher fructose and glucose levels owing to increased expressions of genes enriched in photosynthesis (and related pathways) and sugar-related pathways. Consistent with these expressions, the higher expression of pectinesterase (PE) and pectate lyase (PL) in GX74 as compared to XY15 indicates that more sugar resources are being allocated in GX74 for cell wall modification for cold stress acclimatization. It is also known that PE activity increases in oil seed rape to acclimatize to cold stress [68]. In addition, a number of XTH genes expressed between the two genotypes could account for variation in cell wall integrity during and after cold stress. The XTH family is well known to play an important role in cell wall reconstruction and stress resistance in plants [69]. For example, XTH9 has been demonstrated to influence freezing tolerance after cold and sub-zero acclimation in Arabidopsis [70]. The increase in pyruvic acid and consistent upregulation of PK in GX74, and the contrasting results in XY15, also supplement the above statement. Pyruvate accumulation is one of the key metabolomic reprogramming events in plants in response to cold and other abiotic stresses [71]. The higher sugar content (fructose and glucose), together with the upregulation of a relatively higher number of XTHs and expansins in GX74 as compared to XY15, could be a reason for better cold adaptability in GX74 since a mutation in XTH (*AtXTH19*) increases cold sensitivity in Arabidopsis [70]. Similarly, overexpression of expansins in plants e.g., tobacco [72], petunia [73], and zucchini [74] resulted in improved cold stress (and oxidative stress). Both the upregulated XTHs and expansin are promising candidates for gene manipulation and testing of how they change the cold sensitivity of brassicas. Other than these factors, the increased expression of boron transporter in cold stress also indicates cell wall changes. Boron is important for cell wall structure due to its role in cross-linking of pectin polysaccharides and pectin assembly in the cell wall [75]. Taken together, cold stress tolerance in GX74 can be attributed to the increased expression of genes involved in photosynthesis, antenna proteins, cell wall modification, and chlorophyll biosynthesis, which in turn affect the higher sugar contents.

### 4.2. Differential ROS Scavenging in GX74 and XY15 Is Related to Cold Stress Tolerance

Cold stress causes changing of membrane lipids from a liquid to a gel state, thus affecting the membrane permeability, electrolyte leakage, and loss of ion homeostasis. The damage to membrane lipids (lipid peroxidation) and destruction of proteins is induced by ROS. We observed a non-significant increase in MDA content (Figure 2) in cold-stressed GX74 as compared to XY15, and a relatively higher decrease in ROS (as evident from H_2_O_2_ levels in Figure 2). These observations indicate lipid peroxidation is lower in GX74 (compared to XY15) when challenged with cold stress. This is consistent with the observed relatively higher lipid and lipid-like metabolites in XY15. These observations, together with the ruptured mesophyll cell membranes, indicate that XY15 experiences relatively higher membrane injury than GX74. The higher reduction in ROS in GX74 can be understood from the higher expressions of POD, GPx, APX, and CAT genes (Figure 5) based on their known roles in ROS scavenging [76,77]. These results are consistent with an earlier report in soybean, where ROS reduction was associated with the increased activities of antioxidant enzymes [78]. Additionally, the upregulation of several genes characterized as oxidoreductases, e.g., NADPH oxidase and expansins [49,74], is also consistent with the differential ROS-scavenging potentials and cold stress responses of both XY15 and GX74. These ROS are generated in response to signaling (e.g., Ca^2+^ signaling) and cold perception. In this regard, the higher expressions of Ca^2+^ uniporter and Ca^2+^ proteins in GX74 indicate better Ca^2+^ transport across the mitochondrial membrane [79] and distribution of Ca^2+^ in cells [80]. Furthermore, the upregulation of NHX in GX74 indicates that this genotype activates genes that help to maintain ion homeostasis [81]. In addition to the above mentioned ways through which GX74 might have better ROS-scavenging ability, the expression changes in electron transfer chain-related genes can also be a possible strategy to scavenge ROS [82]. The higher expressions of several mitochondrial electron transfer chain-related genes/proteins, i.e., COX2, cyt-b5, cyt-b561, cyt-b6/f4, and CO2, indicate the possibility that mitochondrial function induces the cold stress responses (genes/proteins) [83], which help GX74 perform better in cold stress compared to XY15. These results are consistent with the report that defects in mitochondrial complexes of the electron transfer chain impair cold-regulated gene expression in Arabidopsis [84].

### 4.3. Abscisic Acid and Auxin-Responsive Genes Are Activated in Response to Cold Stress

Phytohormone signaling also plays significant roles in cold stress responses. Auxin plays essential roles in regulating almost all aspects of plant growth and development. Intracellular auxin transport is affected by cold stress and hence the auxin-responsive genes are affected [85]. The up- and downregulation of a relatively higher number of auxin-responsive genes (GO:0009733) in GX74 (Figure 7) suggests that auxin perception and cellular responses to auxin increased in this genotype as a result of cold stress treatment. Though these responses were also present in XY15, the lower TPM values, as well as a smaller number of DEGs, indicate relatively lower auxin perception and respective cellular responses in XY15. On the other hand, the upregulation of the four ABA responsive genes in both XY15 and GX74 indicates that ABA perception increased in response to cold stress [85], as found in other plants, e.g., bermudagrass [86]. This is consistent with the exclusive upregulation of ABF3 in GX74 (Figure 7). Earlier studies have indicated that ABFs are activated in response to cold stress and are regulated by ICE1 [87]. Similarly, the upregulation of a higher number of AP2-type TAs in GX74 as compared to XY15, in response to cold stress, indicates relatively more cold-responsive genes are active in the former genotype. The AP2-type TAs (CBFs) are an important component of the ICE-CBF-COR pathway that elicits freezing tolerance [53]. Taken together, the GX74’s cold stress tolerance can be due to the higher expressions of a larger number of DEGs associated with the auxin and ABA signaling and ICE-CBF-COR pathway genes.

### 4.4. XY15 and GX74 Exhibit Differential Lipid Metabolism in Response to Cold Stress

Among several strategies, plants also increase lipid biosynthesis and remodel their lipidome [88]. The 3-ketoacyl-CoA synthases were downregulated in both genotypes in response to cold stress; three in XY15 and five in GX74, whereas those involved in fatty acid metabolism were upregulated in GX74 exclusively (Figure 5). Interestingly, the fatty acid degradation-related genes were also exclusively downregulated in GX74 (Figure 5), which is consistent with the detection of a relatively lower number of DAMs classified as lipids. These suggest that GX74 activates the fatty acid metabolism-related genes. The upregulation of fatty acid metabolism genes is also consistent with the earlier reports that cold stress induces lipid remodeling [89].

Other than lipids, phytohormones, and the ICE-CBF-COR pathway, our study also highlighted that cold induced arachidonic acid and magnoflorine biosynthesis in GX74. These results are interesting since arachidonic acid has been shown to increase light-use efficiency in microalgae under chilling stress [90]. Thus, our results highlight a possible interaction between better photosynthetic efficiency and arachidonic acid. Such results have been observed in banana, where arachidonic acid was reported to mitigate chilling injury in cold-stored banana fruit [91]. Research has shown that magnoflorine is a strong antioxidant; thus, induction of magnoflorine biosynthesis in GX74 is an indication of reduced lipid peroxidation. This proposition is based on the earlier work where magnoflorine was reported as an inhibitor of lipid peroxidation and a radical scavenger [92]. Nevertheless, these two compounds should be tested further for their roles in cold tolerance in rapeseed.

### 4.5. Cold Stress Induces Large-Scale Transcriptional Activity in GX74 Compared to XY15

Finally, the overall higher number of DEGs in GX74 indicates relatively higher transcriptional activity, particularly after cold stress treatment. This transcriptional activity is directly associated with the differential regulation of a relatively higher number of TFs. Furthermore, the higher number of upregulated TFs in both genotypes indicates that rapeseed induces a large number of TFs to regulate the cold stress responses (Table 2). These TFs are part of both the signaling and cold-responsive genes/pathways, which function as key players in the regulation of cold stress responses [93].

## 5. Conclusions

**Figure 9:**
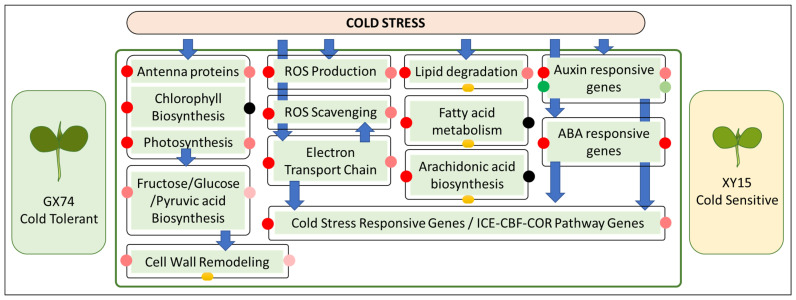
Contrasting cold stress responses of GX74 and XY15 cotyledons as revealed by full-length transcriptome analyses. The red, green, and black dots show the up-, down-, and not regulated genes within each pathway. The reduction in red and green color intensity of the dots indicates a higher and lower number of DEGs involved in each pathway, respectively. The dark yellow circles indicate the metabolites were differentially accumulated related to those pathways.

This study concludes that the cotyledons of XY15 experience relatively greater cold injuries compared to GX74 as observed by TEM. The GX74, a cold-tolerant rapeseed genotype, had higher fructose, glucose, pyruvic acid, and proline contents compared to XY15 when subjected to cold stress. We found that the higher expressions of genes enriched in photosynthesis, antenna proteins, chlorophyll biosynthesis, sugar biosynthesis/metabolism, and pyruvic acid metabolism in GX74 as compared to XY15 are consistent with the fructose and glucose contents. GX74 had a higher number of genes upregulated that were associated with cell wall remodeling. A relatively higher number of DEGs were activated in GX74 for ROS production, as well as ROS scavenging, in response to cold stress as compared to XY15. The upregulation of a larger number of genes involved in the mitochondrial electron transfer chain can also be a possible explanation for GX74’s cold tolerance. Lipid degradation genes were upregulated in both genotypes’ cotyledons; however, fatty acid metabolism genes were only upregulated in GX74. The accumulation of magnoflorine and arachidonic acid in GX74 can also be a reason for its cold tolerance ability. Finally, there were a larger number of upregulated genes in GX74 associated with auxin and ABA responses, as well as the ICE-CBG-COR pathway. Thus, GX74 can possibly survive cold at the seedling stage due to the relatively higher expression of genes associated with the above-mentioned pathways (Figure 9).

## Figures and Tables

**Table 1:** List of primers used for qRT-PCR analysis of *B. napus* genes in GX74 and XY15 cotyledons in response to cold stress.

Gene	Annotation	Forward Primer	Reverse Primer
*BnaAnng18280D*	Calcium uniporter protein	CTGAAGGTGCCGGTTAGGG	CATCGGAGGTGAAAGTCCGT
*BnaAnng34260D*	Dehydration responsive element binding protein	CTGACGTGTCCCTTTGGAGT	ACATTCACACTCAGCTTCCACA
*BnaA06g16150D*	Peroxidase	TGTTATCTCAGTATGTGGCTACCAA	AGAACGTAAAATTACAAAGCACACA
*BnaC08g19360D*	Catalase	GATCCATATCGCGTGGTCGT	TCAAGATTCTCTTTTGAAGTCGTCG
*BnaA07g24600D*	3-ketoacyl-CoA synthase/Fatty acid elongation	AACGTCTTCAAGTACGGTTTGTTT	ACCTCCATTTCCCAATCCCCT
*BnaC03g44110D*	Chlorophyll a-b binding protein, chloroplastic	ATCACTTGGCGGATCCTGTG	TCAGAGGCCACATATCCATTCA
*BnaA04g12130D*	Fructose-bisphosphate aldolase/Amino sugar and nucleotide sugar metabolism	AGCTTGAGCTTGATTTTGGTGT	TCAACTCTCATTGGCCGGTT
*BnaC02g10080D*	UDP-glucuronate decarboxylase/Amino sugar and nucleotide sugar metabolism	AAGCCGATCTCTCTTCTCTTATTCT	CCGCCATTTGCTTTGCTGAG
*BnaA08g25690D*	Xyloglucan endotransglucosylase/hydrolase	TGCAGCAATGGATTGGGCTA	TGAACTCAGCACTCAGCAGG
*BnaA10g01400D*	Pectinesterase	TTACATGGCCCGGTTACCAC	CTGTTAGGTTTGTGCCGCTG
*BnaA02g23870D*	Pectin acetylesterase	CAAGTGTAATGGTGTTAGCCGT	CACTCCTGAGCCAGATCCTT
*BnaA09g21570D*	Glutathione peroxidase	ATTTCGAGATGGCTGCTGCT	TCGCGTCCTTGACTGTGAAA
*BnaA03g53120D*	L-ascorbate peroxidase	AAGAGCCACGAAGCAAAAGA	CGTTGTAATGAAACCGTAACGC
*BnaC02g20560D*	Ribose-5-phosphate isomerase	AGGCGAAGCTAAGGGTTAAGA	GGCAACCTCAATATCGCCTC
*BnaA06g02800D*	HKL1	TCTGCTGGAAAGGCGGTAAT	ACGCCAACCTAACAAATTCCT
*ACTIN2*	ACTIN2	CTGGATTCTGGTGATGGT	GCTTCTCCTTGATGTCTCT

**Table 2:** Summary of major transcription factor families detected among the differentially expressed genes in the two contrasting genotypes of rapeseed.

TF	Genotype ^a^	DEGs			
		Total	Downregulated ^b^	Upregulated ^c^	Common ^d^
B3	XY15	6	1	5	4
	GX74	19	7	12	
bHLH	XY15	31	12	19	10
	GX74	100	51	49	
bZIP	XY15	19	8	11	6
	GX74	26	18	8	
C2H2	XY15	26	2	24	15
	GX74	48	8	40	
ERF	XY15	69	10	59	48
	GX74	139	37	102	
FAR1	XY15	20	9	11	4
	GX74	28	8	20	
MAD	XY15	20	8	12	10
	GX74	32	14	18	
NAC	XY15	29	2	27	19
	GX74	64	15	49	
WRKY	XY15	19	2	17	7
	GX74	29	6	23	

^a^ Cold-sensitive genotype (XY15) and cold-tolerant genotype (GX74). ^b^ Downregulated means genes expressed higher under control (CK) than after cold stress (18 h) (LW). ^c^ Upregulated means genes expressed higher under LW than CK. ^d^ Commonly detected TFs in both genotypes.

## Data Availability

Data is contained within the article and Supplementary Materials.

## Supplementary Materials

The following supporting information can be downloaded at: https://www.mdpi.com/article/10.3390/plants13162212/s1, Table S1: Differentially accumulated metabolites in GX74 and XY15 cotyledons before and after cold stress treatment; Table S2: Statistics of full-length sequence data of cotyledons of two contrasting genotypes of *Brassica napus* L. (cold-sensitive genotype, XY15, and cold-tolerant genotype, GX74). Analyses were conducted on 12 libraries (2 genotypes × 2 conditions [before cold stress (CK) and after cold stress (LW)] × 3 biological repeats (1,2,3)]; Table S3: A. Four hundred and sixty-one unique differentially expressed genes detected in cold-sensitive genotype (XY15) under control and after cold stress (18 h) in the cotyledons of *Brassica napus* L. B. One thousand, four hundred and eighty-one unique differentially expressed genes detected in cold-tolerant genotype (GX74) under control and after cold stress (18 h) in cotyledons of *Brassica napus* L. C. Five hundred and forty-five differentially expressed genes detected in both cold-sensitive genotype (XY15) and -tolerant genotype (GX74) under control and after cold stress (18 h) in the cotyledons of *Brassica napus* L; Table S4: Major transcription factor (TF) families and the profiles of their differentially expressed genes detected in cold-sensitive genotype (XY15) and cold-tolerant genotype (GX74) under control (CK) and after cold stress (18 h) (LW) in the cotyledons of *Brassica napus* L; Table S5: A. Gene ontologies of differentially expressed genes detected in cold sensitive genotype (XY15) under control and after cold stress (18 h) of *Brassica napus* L. cotyledons. B. Gene ontologies of differentially expressed genes detected in cold tolerant genotype (GX74) under control and after cold stress (18 h) of *Brassica napus* L. cotyledons; Figure S1: Gene ontology (GO) pathway enrichment in cold-sensitive (XY15) and -tolerant (GX74) genotypes under control (CK) and after cold stress (18 h) (LW) in the cotyledons of *Brassica napus* L. A. XY15 (CKvsLW). B. GX74 (CKvsLW). *x*-axis indicates the number of differentially expressed genes and *y*-axis indicates GO. The colors represent *p.* adjusted; Figure S2: Highly expressed genes in the cotyledons of GX74 and XY15 genotypes before (CK) and after cold stress treatment (LW). A. Downregulated and B. upregulated in XY15LW compared to XY15CK. C. Downregulated and D. upregulated in GX74LW compared to GX74CK. The heatmaps represent log2 fold change values; Figure S3: Pearson correlation between the expression levels of 21 genes based on qRT-PCR and RNA-seq methods.
