# Supplementary figures and images for: Comparative Metabolome and Transcriptome Analysis of Rapeseed (Brassica napus L.) Cotyledons in Response to Cold Stress
Source: Plants (Basel). 2024 Aug 9;13(16):2212. doi: 10.3390/plants13162212 (PMC11360269; doi:10.3390/plants13162212)

## Slide 1
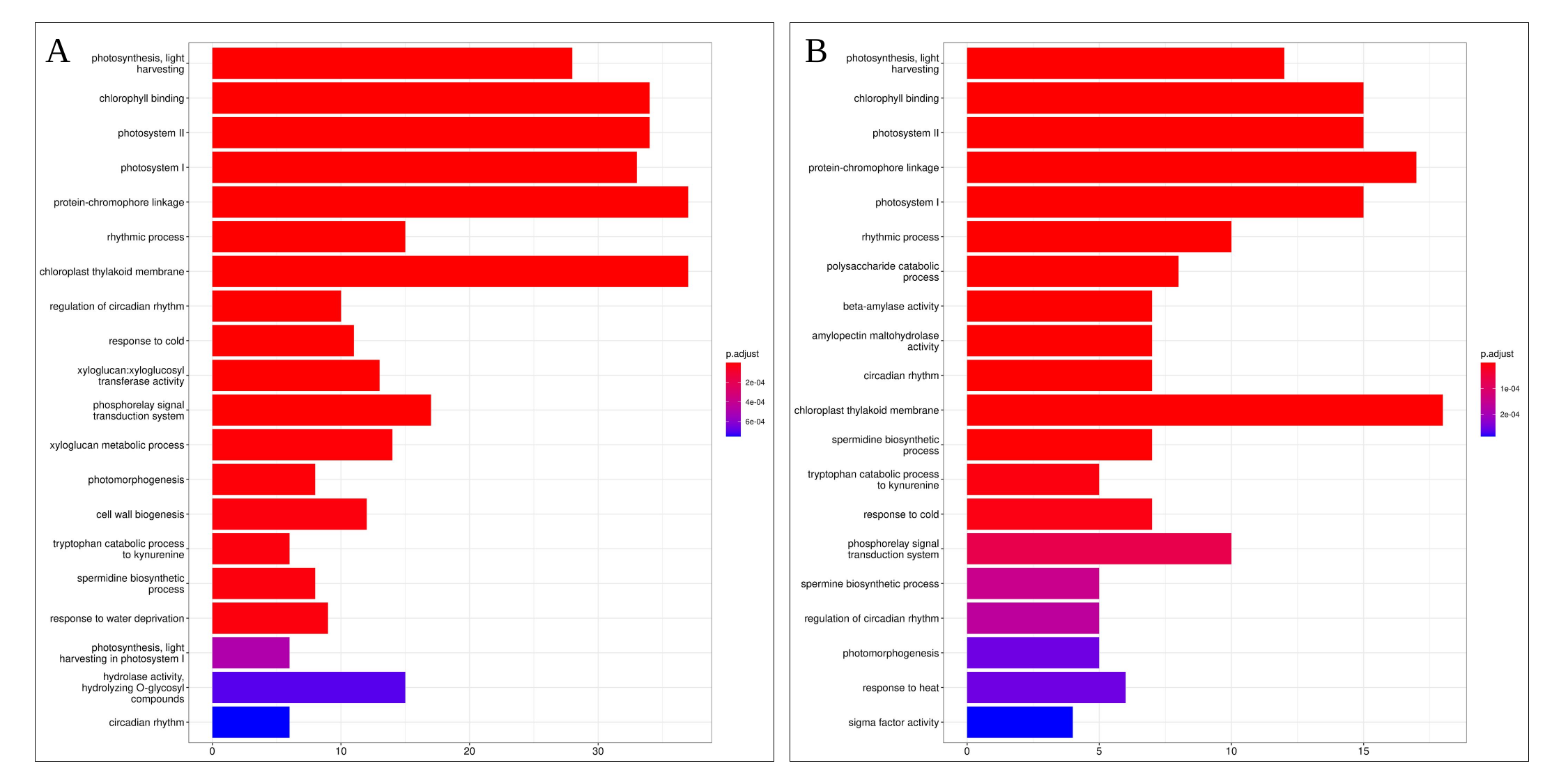

B
A

Supplement: Supplementary file 1 [file plants-13-02212-s001.zip › Figure S1V2.pptx]

## Slide 1
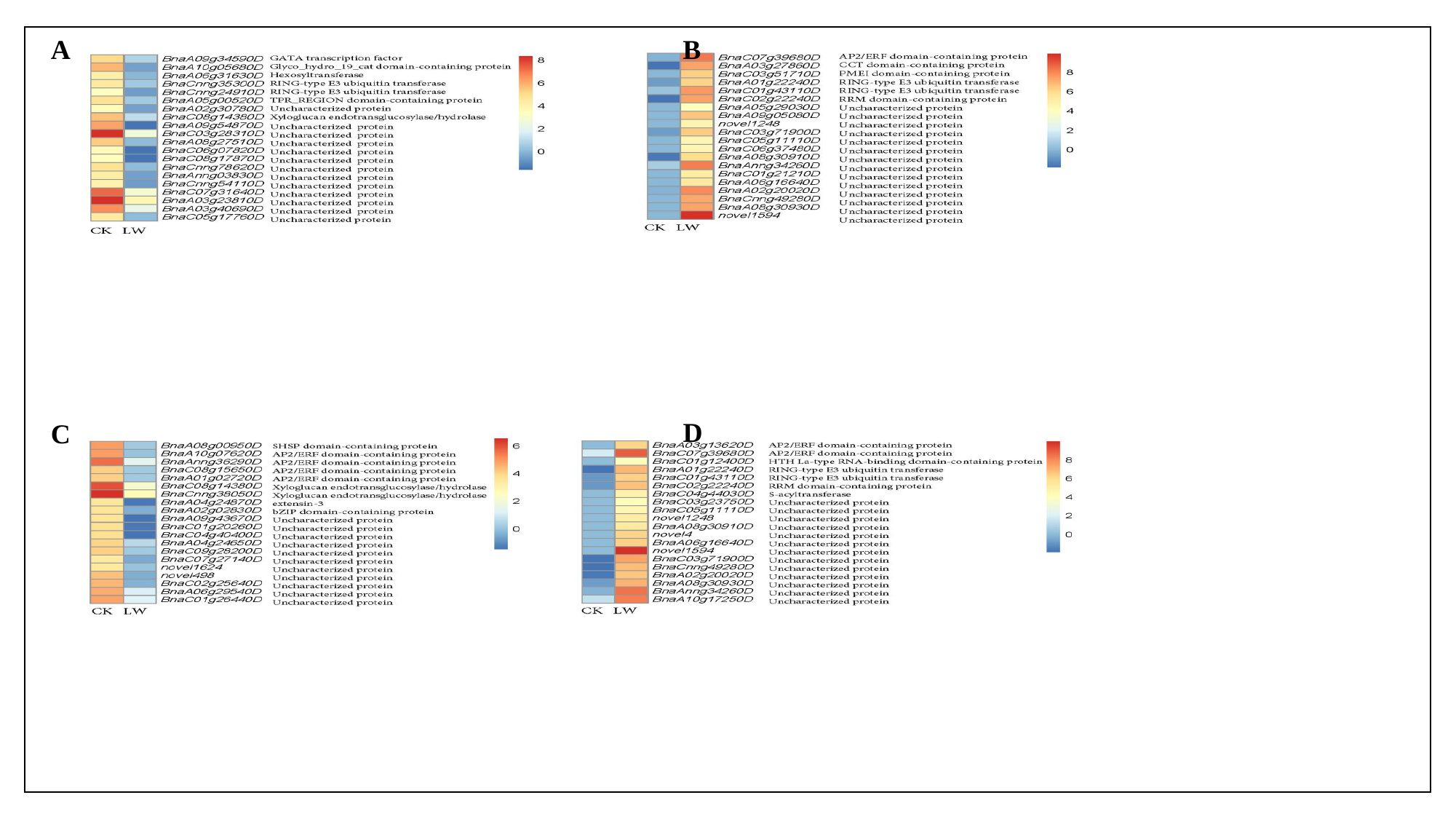

A
B
D
C

Supplement: Supplementary file 1 [file plants-13-02212-s001.zip › Figure S2V2.pptx]

## Slide 1
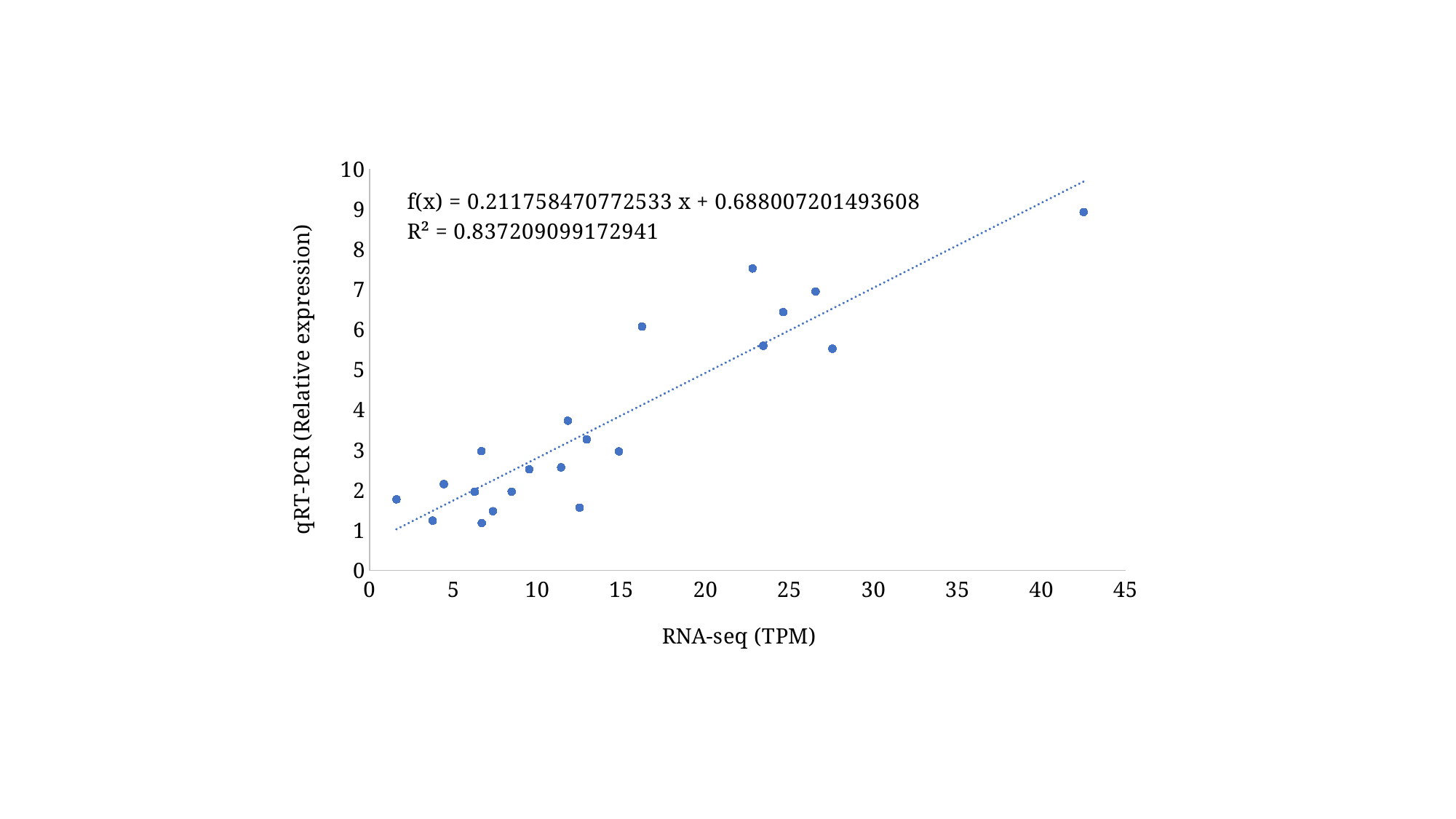

### Chart
| Category | |
|---|---|

Supplement: Supplementary file 1 [file plants-13-02212-s001.zip › Figure S3.pptx]
